# Six-Year Nitrogen–Water Interaction Shifts the Frequency Distribution and Size Inequality of the First-Order Roots of *Fraxinus mandschurica* in a Mixed Mature *Pinus koraiensis* Forest

**DOI:** 10.3389/fpls.2017.01691

**Published:** 2017-09-26

**Authors:** Cunguo Wang, Zhenzhen Geng, Zhao Chen, Jiandong Li, Wei Guo, Tian-Hong Zhao, Ying Cao, Si Shen, Daming Jin, Mai-He Li

**Affiliations:** ^1^College of Agronomy, Shenyang Agricultural University, Shenyang, China; ^2^Swiss Federal Research Institute WSL, Birmensdorf, Switzerland

**Keywords:** first-order roots, *Fraxinus mandschurica*, frequency distribution, Gini coefficient, Lorenz asymmetry coefficient, nitrogen–water interaction, root traits, size inequality

## Abstract

The variation in fine root traits in terms of size inequality at the individual root level can be identified as a strategy for adapting to the drastic changes in soil water and nutrient availabilities. The Gini and Lorenz asymmetry coefficients have been applied to describe the overall degree of size inequality, which, however, are neglected when conventional statistical means are calculated. Here, we used the Gini coefficient, Lorenz asymmetry coefficient and statistical mean in an investigation of *Fraxinus mandschurica* roots in a mixed mature *Pinus koraiensis* forest on Changbai Mountain, China. We analyzed 967 individual roots to determine the responses of length, diameter and area of the first-order roots and of branching intensity to 6 years of nitrogen addition (N), rainfall reduction (W) and their combination (NW). We found that first-order roots had a significantly greater average length and area but had smaller Gini coefficients in NW plots compared to in control plots (CK). Furthermore, the relationship between first-order root length and branching intensity was negative in CK, N, and W plots but positive in NW plots. The Lorenz asymmetry coefficient was >1 for the first-order root diameter in NW and W plots as well as for branching intensity in N plots. The bimodal frequency distribution of the first-order root length in NW plots differed clearly from the unimodal one in CK, N, and W plots. These results demonstrate that not only the mean but also the variation and the distribution mode of the first-order roots of *F. mandschurica* respond to soil nitrogen and water availability. The changes in size inequality of the first-order root traits suggest that Gini and Lorenz asymmetry coefficients can serve as informative parameters in ecological investigations of roots to improve our ability to predict how trees will respond to a changing climate at the individual root level.

## Introduction

The first-order roots (the distal roots) of the fine root system play a key role in nutrient and water absorption because they are located in the root hair zone (He et al., 2005), which is the metabolic hotspot for exudations that mobilize less mobile nutrients (Pregitzer, 2003) and is the point of association with symbiotic mycorrhizal fungi (Guo et al., 2008). Global change, including increasing nitrogen (N) deposition and changing precipitation patterns, has a significant impact on soil nutrient and water conditions of forest ecosystems worldwide (Liu et al., 2013; Brunner et al., 2015; Tietjen et al., 2017). The urgent issue from an ecological perspective is determining the implications of different degrees of soil nitrogen and/or water availability for the functionality of the tree root system (Pregitzer et al., 1993; Wang et al., 2012; Liu et al., 2015). Recently, considerable advances have been made to better understand the responses of fine roots, specifically first-order roots, to nitrogen and/or water availability in forest ecosystems (Lim et al., 2015; Liu et al., 2015; McCormack et al., 2017). The results of fine root traits and their general response trends are typically depicted using means or medians and the associated statistics, such as standard errors or standard deviations (Lim et al., 2015; Liu et al., 2015). These metrics simply eliminate the variation at the individual root level based on the assumption that the variation is experimental error only (Trewavas, 2003). Accordingly, changes in mean values are only a composite population response to environment changes and may conceal important information about the variability among individual roots (Zieschang and Sievers, 1991; Amzallag, 2001; Trewavas, 2003). For instance, the gravi-responding trajectories of individual roots are too complex to be summarized using statistical mean values (Zieschang and Sievers, 1991). The key part of responses in terms of growth and development of the plant root system to environment changes may be hidden using classical methods that compare mean values (Amzallag, 2001). It may be important to pay attention to the ecological implications of individual root variation occurring within the fine root system, an important mechanism for adapting to the drastic variation in water and nutrient supplies occurring with global environment change (Forde, 2009; Russell et al., 2014; Zadworny et al., 2016).

Size inequality (variability in the size of individuals) prominently contributes to our understanding of root structural diversity, a vital factor influencing many ecological functions in forest ecosystems (García, 2006; Metsaranta and Lieffers, 2008). Size inequality in tree height makes it possible to produce the greater packing densities of different tree canopy heights and thus to enhance the higher aboveground light capture and solar utilization efficiency within a forest ecosystem (Zhang and Chen, 2015). The plant root system is thought to be modular (Majdi et al., 2001) and often exhibits complex branching patterns to ensure effectiveness in resource exploitation (Trewavas, 2003; Kong et al., 2014; Liese et al., 2017). The question of how fine roots cope with variable soil conditions can be addressed at the individual root level (Zieschang and Sievers, 1991; Forde, 2009). Unfortunately, the characteristics of the distribution and the variability among root individuals are not well-known (Eissenstat and Achor, 1999). The information depicting the size inequality of tree traits has not been incorporated into forest ecosystem models that employ plant functional trait data (Russell et al., 2014). Therefore, approaches that characterize variability at the individual root level are urgently needed for better comprehension of the nature of the collective responses and adaptations of root individuals in a changing world (Trewavas, 2003; He et al., 2005).

The Lorenz curve (Gini coefficient) is widely applied in economics to graphically describe the degree of inequality in the distribution of wealth or income in societies (Lorenz, 1905). Theoretically, the Gini coefficient is distinct from the conventional statistical standard deviation and standard error (He et al., 2005). It ranges between zero and one in value to reflect the degree of deviation from a situation where all individuals are equal, whereas standard deviations or errors indicate the extent to which individual observations in a data set are dispersed around the mean (He et al., 2005). Thus, the Gini and Lorenz asymmetry coefficients may be used to compare inequalities of populations with different means (Weiner and Solbrig, 1984). For example, the Gini and Lorenz asymmetry coefficients have been applied to quantify the size inequality of plant functional traits, such as biomass and fecundity in several previous studies (Weiner and Solbrig, 1984; Damgaard and Weiner, 2000). The incorporation of a complete functional trait profile including the Gini coefficient has also been used to comprehend large-scale patterns of forest ecosystem structure and production (Metsaranta and Lieffers, 2008; Russell et al., 2014).

The Gini and Lorenz asymmetry coefficients may be used to deepen our understanding of the overall degree of size inequality associated with root traits in fine root systems and to give insights into the relationships between root inequality and distribution patterns (Lieffers and Titus, 1989; He et al., 2005; Magura et al., 2006). The optimal photoassimilate allocation within a tree or in a root system will not only minimize the competition for resources but also maximize the forage efficiency (Hutchings and De Kroon, 1994; Forde, 2009). Changes in soil nutrient or water availability may stimulate the growth of some root individuals but not others, which will lead to changes in the shape of size (e.g., root length) frequency distribution in a fine root system, and thus result in changes in Gini and/or Lorenz asymmetry coefficients (Lieffers and Titus, 1989). In this study, we primarily focused on the frequency distribution of root individuals, a variable that plays an important role in generating variation among root individuals to cope with the instability of forest soil nitrogen and water contents (He et al., 2005; Forde, 2009). We calculated the statistical means of the first-order root length, diameter, area and branching intensity, as well as the Gini and Lorenz asymmetry coefficients of size inequality of the root traits, using data from 967 first-order root individuals of *Fraxinus mandschurica*, to estimate the responses of tree roots to 6 years of simulated increased nitrogen deposition and decreased rainfall in a mixed mature *Pinus koraiensis* forest on Changbai Mountain, China. We expected that Gini and Lorenz asymmetry coefficients would yield insights into the response patterns of the first-order root traits of *F. mandschurica* to changes in soil nitrogen and/or water availability.

## Materials and methods

### The study forest

The experiment was conducted within the Changbai Mountain Nature Reserve (42° 24′N, 127° 47′E) in Jilin province in northeastern China. The Changbai Mountain Nature Reserve has a temperate, continental climate. In this region winters are long and cold, whereas summers are short and cool. The mean annual and growing season temperatures are approximately 3.5° and 15.0°C, respectively. The highest (20.5°C) and lowest (–16.5°C) monthly mean temperatures occur in August and January, respectively. The mean annual precipitation (1982–2012) is approximately 715 mm, 70–80% of which occurs during the growing season between May and October (Zheng et al., 2017). Rainfall data collected at the Forest Ecosystem Research Station of Changbai Mountain at 738 m a.s.l. show that the precipitation in drought years, such as 1985, 1997, 1999, 2001, and 2003 is about 30% less (715 mm) than the long-term mean annual precipitation in the last 30 years (Figure S1). In addition, the total (wet and dry) nitrogen deposition of Jilin province yearly increases by 13.79 kg N ha^−1^, which is higher than the national average level (12.89 kg N ha^−1^ year^−1^) (Lü and Tian, 2007). *F. mandschurica* in the mixed mature *P. koraiensis* forest is the dominant broad-leaf tree species, with the mean canopy height of 24.1 m and diameter at breast height of 69.2 cm. The soil bulk density of this study forest is 0.35 g cm^−3^ at 0–10 cm soil depth and 0.68 g cm^−3^ at 10–20 cm soil depth. The soils classified as Eutric cambisol (FAO classification) are developed from volcanic ash.

### Design of experiment

In September 2009, six 50 × 50 m plots (three reduced precipitation plots and three control plots) were randomly established in the mixed mature *P. koraiensis* forest. There was a >20 m buffer strip between any two plots. Thirty percent of the plot area was covered with high-light-transmittance (transparency 95%) polycarbonate V-shaped panels to intercept 30% of the throughfall, or approximate 215 mm year^−1^. A distance of about 1 m from the V-shaped panels to the ground was maintained to keep normal air flow (Figure S2). Each of these six plots was split into two 25 × 50 m subplots with an iron sheet inserted 50 cm deep into the ground. One subplot received additional nitrogen and the other was simultaneously supplied with the same amount of water. Four treatments with three replicates per treatment were developed: control treatment (CK, ambient rainfall without nitrogen addition), nitrogen addition treatment (N), rainfall reduction treatment (W) and the nitrogen addition combined with rainfall reduction treatment (NW). The applied nitrogen level of 50 kg N ha^−1^ year^−1^ was about two times the annual total nitrogen deposition in this area (23 kg N ha^−1^ year^−1^) (Lü and Tian, 2007). NH_4_NO_3_ was diluted in 40 L of water and then sprayed onto the forest floor with a backpack sprayer. The control plots including W treatment plots were sprayed with 40 L of water each month during the entire growing season (May to October) to avoid an effect of the treatments on soil moisture.

### Root collection and trait measurement

In August 2015, after 6 years of nitrogen addition and water reduction, soil cores (5 cm internal diameter by 20 cm depth) were collected at five random locations from each plot. The five soil cores from each plot were pooled into one subsample and placed immediately into coolers containing ice. In the laboratory, all samples were kept at –4°C until processing, which was completed within about 1 week. The living *F. mandschurica* roots were manually picked from soil samples in Petri dishes using forceps and then washed under running water. The roots were dissected into first- and second-order roots according to the protocols described by Pregitzer et al. (2002). Root branching intensity is measured as the number of first-order roots per centimeter of second-order roots. In all, we obtained 967 and 112 first- and second-order roots, respectively. The collected roots were scanned with an Epson Expression 10000XL scanner. Images were analyzed with WinRhizo (Regent Instruments, Inc., Québec, Canada) to determine the count, length, diameter and area of first- and second-order roots.

### Statistical analyses

Owing to non-normality in the data set, non-parametric tests (*kruskal.test* function) were chosen to examine the treatment effects. We used a TukeyC test to investigate differences among treatments, followed by a least significant difference *post-hoc* test when *p* < 0.05. A Kolmogorov-Smirnov test (*ks.test* function) was used to test the differences in distribution for first-order root length, diameter, and area among treatments. Simple correlations of first-order root length, diameter and area with branching intensity were determined for each treatment using Pearson correlations (*cor* function). In addition, we used two-way ANOVAs (*aov* function) to test the effects of nitrogen addition, water reduction and possible interactions between these treatments for each root trait after log-transforming the variables.

In this study, the term “size” was defined as the measured values of root length, diameter and area for individual first-order roots. We calculated the following two size inequality metrics: Gini coefficient and Lorenz asymmetry coefficient of first-order root length, diameter and area for each treatment. The Gini coefficient is based on the Lorenz curve, which graphically represents a population distribution; a minimum value of zero indicating equal size of all individuals and a maximum value of one indicating maximum inequality within the population (Weiner, 1985; Damgaard and Weiner, 2000; McGown et al., 2015). However, the Gini coefficient does not contain all the information in the Lorenz curve because different Lorenz curves can hold the same Gini coefficient (Weiner and Solbrig, 1984). Thus, the Lorenz asymmetry coefficient is calculated to evaluate whether the observed size inequality is primarily caused by large or small individuals (Damgaard and Weiner, 2000; Magura et al., 2006). When LAC = 1, the Lorenz curve of the population is symmetric; when LAC > 1, most of the size inequality within the population is the result of the largest individuals; when LAC < 1, the size inequality demonstrated within the population is mainly due to a relatively large number of small individuals (Damgaard and Weiner, 2000; Weremijewicz and Janos, 2013). R code developed by Buckley and Damgaard (2012) was used to calculate Gini and Lorenz asymmetry coefficients.

## Results

The average values of first-order root length (5.89 ± 3.55 mm) and area (3.28 ± 2.59 mm^2^) in NW treatment plots were 26 and 32% greater, respectively, than those (4.67 ± 3.08 mm and 2.48 ± 1.78 mm^2^) in CK treatment plots. By contrast, there were no significant differences in second-order root length or area across all treatments (Table 1). Compared with CK, N treatment significantly decreased first-order diameter, while second-order root diameter was 1.3-fold larger in W treatment plots than in N treatment plots (Table 1). Root length, diameter and area consistently increased from first- to second-order roots over all treatments (Table 1). Furthermore, second-order roots had fewer first-order roots (lower branching intensity) in NW treatment plots than in W treatment plots (Table 1). Significant two-way interactions (NW) were observed for first-order root length, diameter and area, for second-order root length and for branching intensity (Table S1).

**Table 1:** The effects of nitrogen addition (N), rainfall reduction (W) and their interactions (NW) on first- and second-order root lengths (FL and SL), diameters (FD and SD) and areas (FA and SA), as well as effects on branching intensity (BI) of *F. mandschurica*.

**Treatments**	**FL (mm)**	**FD (mm)**	**FA (mm^2^)**	**SL (mm)**	**SD (mm)**	**SA (mm^2^)**	**BI (no. cm^−1^)**
CK	4.67 ± 3.08^b^ (399)	0.17 ± 0.04^a, b^ (399)	2.48 ± 1.78^b^ (399)	22.51 ± 5.54^a^ (45)	0.25 ± 0.07^ab^ (45)	17.79 ± 7.80^a^ (45)	3.88 ± 1.52^ab^ (45)
N	4.01 ± 2.66^b^ (123)	0.15 ± 0.04^c^ (123)	1.98 ± 1.45^b^ (123)	20.11 ± 8.61^a^ (15)	0.22 ± 0.05^b^ (15)	15.96 ± 9.03^a^ (15)	4.28 ± 1.41^ab^ (15)
W	4.59 ± 3.38^b^ (198)	0.16 ± 0.05^b, c^ (198)	2.41 ± 3.04^b^ (198)	19.91 ± 5.87^a^ (22)	0.28 ± 0.06^a^ (22)	17.42 ± 6.75^a^ (22)	4.61 ± 1.87^a^ (22)
NW	5.89 ± 3.55^a^ (247)	0.17 ± 0.05^a^ (247)	3.28 ± 2.59^a^ (247)	23.83 ± 8.78^a^ (30)	0.25 ± 0.04^ab^ (30)	19.31 ± 9.78^a^ (30)	3.41 ± 1.11^b^ (30)

*The average values ± 1 standard deviations are given. Numbers in parentheses are total numbers of observations. Different letters within a column indicate statistical significance at p < 0.05*.

**Figure 1:**
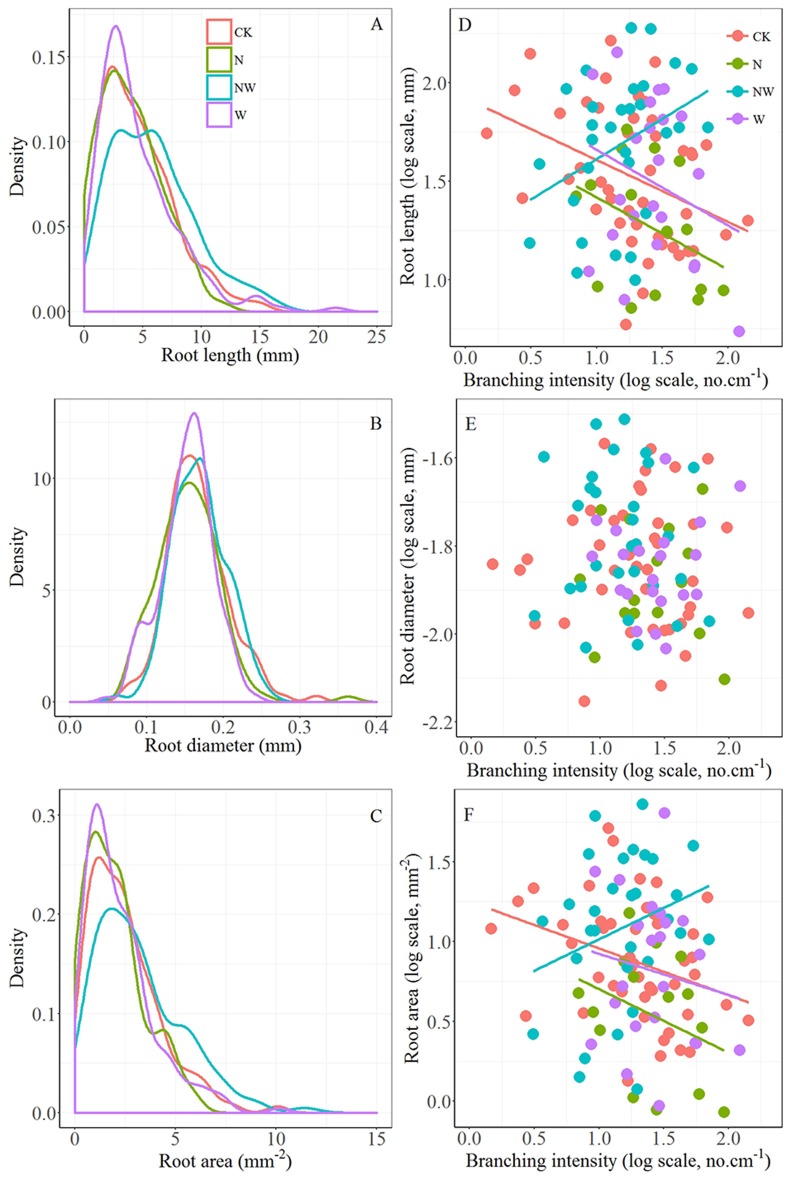
Left column: kernel density estimates of first-order root length **(A)**, diameter **(B)** and area **(C)** for control plots (CK) and those treated with nitrogen addition (N), rainfall reduction (W), or the combination of nitrogen addition and rainfall reduction (NW). The y-axis (density) indicates the abundance of root traits with a given value. Each frequency distribution represents about 399, 123, 247, and 198 first-order roots for CK, N, W, and NW treatment plots, respectively. Right column: relationships between branching intensity and first-order root length **(D)**, diameter **(E)** and area **(F)** of *F. mandschurica*.

First-order length and area in CK treatment plots showed a significant linear decline with increasing branching intensity (*r* = –0.38, *p* < 0.01 and *r* = –0.33, *p* < 0.05, respectively), while first-order length in NW treatment plots increased significantly with branching intensity (*r* = 0.36, *p* < 0.05; Figures 1D, F). Although the relationships were not significant, first-order length and area also showed decreasing trends with increasing branching intensity in N and W treatment plots (Figures 1D, F). First-order root diameter remained stable with branching intensity within all treatment plots (Figure 1E, all tests *p* > 0.05).

In terms of first-order length, the variation in Gini coefficient ranged from 0.33 (NW treatment plots) to 0.38 (N and W treatment plots; Table 2). Lower Gini coefficients of first-order diameter and area but a higher Gini coefficient of branching intensity was observed in CK treatment plots than in other treatment plots (Table 2). For all treatments, the Gini coefficients of first-order root length and area were larger than those of first-order root diameter and branching intensity (Table 2). The Lorenz asymmetry coefficient for first-order root diameter in NW (1.16) and W (1.03) treatment plots and the Lorenz asymmetry coefficient for branching intensity in N (1.08) treatment plots were >1, while this coefficient was <1 in all other cases (Table 2).

**Table 2:** The effects of nitrogen addition (N), rainfall reduction (W) and their interaction (NW) on the Gini and Lorenz asymmetric coefficients of first-order root length (FL), diameter (FD), and area (FA) and of branching intensity (BI) of *F. mandschurica*.

	**Gini coefficient**				**Lorenz asymmetry coefficient**			
**Treatments**	**FL**	**FD**	**FA**	**BI**	**FL**	**FD**	**FA**	**BI**
CK	0.36	0.12	0.38	0.21	0.86	0.91	0.91	0.97
N	0.38	0.14	0.41	0.19	0.83	0.87	0.82	1.08
W	0.38	0.16	0.45	0.16	0.93	1.03	0.99	0.99
NW	0.33	0.16	0.40	0.18	0.83	1.16	0.97	0.89

First-order root length and area did not exhibit a normal distribution (Figure 1). The bimodal frequency distribution of FL in NW treatment plots with peaks at 3.18 and 5.72 mm was significantly different (*p* < 0.001 in all test) from the similar unimodal frequency distributions in CK, N and W treatment plots, which peaked at 2.37, 2.54, and 2.74 mm, respectively (Figure 1A). The distributions of first-order root diameter were normal and did not obviously differ among the treatments (Figure 1B, *p* > 0.05 in all tests). The distribution of area in NW treatment plots also differed markedly from those in CK, N, and W treatment plots (*p* < 0.001 in all tests), and the distributions were skewed strongly to the right for all treatments (Figure 1C).

## Discussion

Given the striking increase in nitrogen deposition combined with a decrease in precipitation in northeastern China (Lü and Tian, 2007), it is imperative to investigate the response of fine root functional traits (e.g., root length, diameter) to long-term changes in soil nitrogen and water availability in forest ecosystems. This is especially true for fine roots, especially first-order roots, because of their vital function in nutrient and water absorption (Pregitzer et al., 1993; Guo et al., 2008). Here, we aimed to understand the responses of first-order root traits to the changes in water and/or nitrogen based on statistical means and the Lorenz curve after 6 years of simulated increased nitrogen deposition and reduced precipitation in a mixed mature *P. koraiensis* forest on Changbai Mountain.

### The responses of first-order root traits to altered nitrogen and water availability based on statistical means

Root diameter is recognized as a key trait in root morphology that can reflect water and nutrient uptake functionality of a root system (Comas and Eissenstat, 2009; Gu et al., 2014). In this study, compared with the CK treatment, the N treatment significantly reduced first-order root diameter, which is in agreement with a study from a *F. mandshurica* plantation (Wang et al., 2007). Moreover, our previous research conducted in the same sites as this study showed a decreasing average diameter of fine roots at the community level after a 2-year N treatment (Guo et al., 2016). It has been demonstrated that roots with smaller average root diameters tend to have less thickening of exodermal walls than thicker roots and are likely to a exhibit higher nutrient absorptive capacity and have lower construction and maintenance costs (Eissenstat, 1991; Pregitzer et al., 1993). Hence, thinner roots may be more advantageous to tree development, given that trees decrease belowground carbon allocation with increasing nutrient availability (Bae et al., 2015). Furthermore, there is an impressive amount of evidence that root diameter also strongly influences root lifespan (Wells et al., 2002; McCormack et al., 2012). Decreasing diameter (indicating higher turnover) of first-order roots in N treatment plots, together with their lower decomposition rates (Fan and Guo, 2010; Goebel et al., 2011), may imply a greater accumulation of *F. mandschurica* root necromass with increasing nitrogen deposition in the experimental temperate forest (Clemmensen et al., 2013).

Trees can enhance root resource absorption not only by producing thinner roots but also by enhancing root length and branching intensity to rapidly exploit resource-rich soil patches, a behavior that has been observed for a number of plant species (Ostonen et al., 2007; Kong et al., 2014). We found that the combination of increased nitrogen with reduced rainfall (NW) significantly enhanced first-order root length, a variable that was not influenced by nitrogen addition (N) or rainfall reduction (W) treatments applied separately (Table 1). It is reasonable to speculate that the larger first-order root area in NW treatment plots is primarily caused by longer length rather than by larger diameter. Therefore, under lower water and higher nitrogen availability conditions, *F. mandschurica* in the mixed mature *P. koraiensis* forest can increase root surface area to increase the volume of soil explored by producing longer roots that have a larger specific root area. Among functional traits, water and nutrient absorption per unit root mass increase with specific root area if area-related absorption rates remain unchanged (Tyree et al., 1998). This result is consistent with results for *Picea abies* L., *P. sylvestris* L., and *Betula pendula* Roth in a previous study, where the specific root area of mycorrhizal roots (namely first-order roots) increased with site fertility along a broad latitudinal gradient (Ostonen et al., 2007).

Root branching intensity, defined as the number of first-order roots per centimeter of second-order roots, is also a key trait governing the volume of soil explored and thus may affect nutrient acquisition (Comas and Eissenstat, 2009; Kong et al., 2014). In this study, we did not observe effects of nitrogen addition (N), rainfall reduction (W), or their combination (NW) on branching intensity of *F. mandschurica* compared with CK. However, the NW treatment evidently had negative effects on branching intensity in comparison with the W treatment (Table 1). The different response patterns of fine roots to nitrogen between ambient and reduced water availability suggest that water availability may play an important role in mediating tree root system responses to increased nitrogen availability in the mixed forest. Soil mineral nitrogen is dissolved in the soil solution, and the presence of water affects soil mineral nitrogen mobility and loss (Harpole et al., 2007). Such an interaction may be important in natural forest ecosystems, such as the mixed mature *P. koraiensis* forest, where fine roots are often subject to multiple covarying resources. Thus, our results suggest that studies of the effects of nitrogen deposition on fine root traits should take into account potential changes in precipitation regime (Harpole et al., 2007; White et al., 2007).

### The responses of size inequality of first-order root traits to changed nitrogen and water availability based on the lorenz curve

Size inequality of individuals exists in most plant populations, and the associated inequality of plant functional traits may have substantial ecological implications (Metsaranta and Lieffers, 2008; Forde, 2009). The variation in first-order root length, diameter and area within a fine root system can be considered a strategy to reduce ecological niche overlap (e.g., root lifespan overlap) and therefore competition between individual roots, resulting in exploration of a greater volume of soil (Pagès et al., 1993; Nadelhoffer, 2000; Pregitzer et al., 2002). This idea can be summarized: plants don't put all their eggs in one basket (Forde, 2009). One approach for achieving this outcome may be through the generation of differentiations in fine root architecture, morphology and/or vertical distribution among individual plants (Bennett et al., 2002; Forde, 2009).

Traditionally, variations in plant quantitative traits have been described and analyzed using the statistical standard deviation and skewness coefficient. In 1984, the Gini coefficient, widely applied in economics, was first introduced in plant ecology to estimate the inequality distribution (Weiner and Solbrig, 1984). Our results of different Gini coefficients clearly demonstrated distinct responses of size inequality of first-order root length, diameter and area and of branching intensity among different treatments (Table 2). Nitrogen addition treatments increased the size inequality (higher Gini coefficients) of *F. mandschurica* first-order root length, diameter and area compared with the CK treatment (Table 2), a response similarly observed in a previous study of *P. contorta* Loudon (Lieffers and Titus, 1989). However, the responses in size inequality of root traits to soil nitrogen availability may be modified by interactions with other environmental variables, such as soil water condition (Table 2). The results reported here suggest that the nitrogen–water interaction has a striking effect on the frequency distribution of first-order root length and area (Figures 1A, C), characterized by a lower branching intensity (Table 1) and a lower Gini coefficient of first-order root length in NW treatment plots (Table 2). The increasing degree of uniformity in first-order root length, as reflected by decreasing inequality (lower Gini coefficient; Table 2), indicates that *F. mandschurica* might enhance the development of shorter roots to make root length more uniform under the combination of greater nitrogen and water availability. Thus, our observations that the responses of roots to the multifactor treatment differed from simple combinations of single-factor responses further indicate a non-additive effect of nitrogen addition and water induced on size inequality of tree fine roots (Niu et al., 2009). In addition, we found that the Gini coefficients of first-order diameter and branching intensity were always less than those of first-order length and area in all treatment plots (Table 2), together with a stable first-order diameter and branching intensity in all treatments (Figure 1D), meaning that root diameter and branching could be characterized as more stable than root length and area (Chen et al., 2013; Kong et al., 2014). Therefore, roots might adapt to changes in soil nitrogen or water through variation (Gini coefficient) of root diameter at individual level (Table 2) while maintaining invariable mean values at the population level (Table 1).

As one of the metrics that describes the shape of the size distribution, the Lorenz asymmetry coefficient is sensitive to minor changes in size distributions and may therefore be helpful in identifying changes in various fine root systems of forest ecosystems. After 6 years of water and nitrogen management, the Lorenz asymmetry coefficient of first-order root diameter in NW treatment plots (1.16) was higher than that in CK treatment plots (0.91; Table 2). The result of a Lorenz asymmetry coefficient of more than one suggests that the first-order roots of *F. mandschurica* exhibit a greater degree of asymmetry (higher Gini coefficient), with a greater proportion of thicker first-order roots in NW treatment plots than in CK treatment plots (Figure 1A). However, the discrepancies in first-order root diameter between NW and CK treatment plots were not observed based on statistic average values of first-order root diameter (Table 1). Thus, the ecological interpretations of patterns of fine root responses to soil environmental changes should include the inequality of root traits by considering the Gini coefficient and Lorenz asymmetry coefficient.

Moreover, there is a large body of studies focused on the effects of individual interactions, such as competition, on size distributions, which indicates that changes in size inequality may often be attributed to an alteration in the competition mode during different root development stages (He et al., 2005; Chu et al., 2009; Forde, 2009; McGown et al., 2015). Competitive interactions between root individuals can be placed along a continuum from completely size-symmetric competition (resource utilization is equal or proportionate to root size) to completely size asymmetric competition (resource utilization is dominated by larger roots) (Schwinning and Weiner, 1998). In general, size asymmetric competition occurs in shoot competition within plants for light. For instance, leaves located at the top of the tree canopy can pre-empt the utilization of light energy by leaves lower in the tree canopy (Schenk, 2006). Size asymmetric root competition involves nutrient and water assimilation (Rewald and Leuschner, 2009). It has been hypothesized that in heterogeneous soils, such as this experimental field (Wang et al., 2010; Xu et al., 2010) the chance of encountering and using soil resource patches may increase nonlinearly with root system size, potentially causing size asymmetric root competition (Schwinning and Weiner, 1998). In this work, we observed larger average values of first-order root length (Table 1) and a higher frequency distribution of longer first-order root length (Figure 1A) in NW treatment plots, where asymmetric competition may occur because of improved chances of acquiring resources conferred by the longer roots (Schenk, 2006). In turn, first-order root length can be expected to increase with increasing branching intensity when competition is asymmetric (NW treatment), whereas first-order length should decrease with increasing branching intensity when competition is symmetric, such as in the CK, N, and W treatment plots (Figure 1D).

## Conclusions

Our work is clearly different from many previous studies that only characterized differences in fine root traits at the population level among species, with little information on how these traits vary at the individual level within the fine root system. We provide the first report of using Gini and Lorenz asymmetry coefficients for evaluating size inequality of *F. mandschurica* first-order root traits in response to nitrogen addition and/or decreased rainfall in the mixed mature *P. koraiensis* forest on Changbai Mountain. Understanding the variation in size inequality in fine root traits can be as informative as the average values when considering effects of the changing global climate. Our results demonstrate that first-order roots of *F. mandschurica* can response to soil nitrogen and water availability in the mean (Table 2) but also in the variance and shape of the distribution (Figure 1), suggesting that the Gini and Lorenz asymmetry coefficients can serve as additional valuable parameters for extracting information not revealed by means alone (He et al., 2005). These findings will improve our understanding of responses of fine roots to simultaneous changes in soil water and nitrogen availability in temperate forest ecosystems. However, as a very first attempt to use the Gini and Lorenz asymmetry coefficients for fine root research, we ignored the effects of the coexisting species *P. koraiensis* on *F. mandschurica* roots. The fine root morphological traits may, indeed, differ in mixed species forests compared to monocultures. For instance, trees that experience intense competition tend to have increasing specific root area or root branching (Fujii and Kasuya, 2008; Xiang et al., 2015), and larger variations of fine root morphological traits (Jacob et al., 2014). Thus, determining the responses of root traits of different tree species (e.g., *P. koraiensis* vs. *F. mandschurica* in this experiment site) to nitrogen-water interaction in that mixed forest will be a vital next step. In addition, given that significant variation among individual roots in morphology and function has important physiological and ecological consequences, it takes far more effort for Gini coefficient than for mean values to thoroughly understand mechanisms of size inequality in root traits (Eissenstat and Achor, 1999). It will be interesting to determine whether the size inequality of tree fine root traits is consistent among different species under uniform and/or contrasting climate environmental conditions.

## Author contributions

CW conceived the ideas; ZG, ZC, SS, and DJ collected the data; CW and JL performed the analysis; CW wrote the first draft; WG, ML, YC, and TZ led the writing of the manuscript. This work has been approved for publication by all co-authors.

### Conflict of interest statement

The authors declare that the research was conducted in the absence of any commercial or financial relationships that could be construed as a potential conflict of interest.
