# Supplementary material for: Six-Year Nitrogen–Water Interaction Shifts the Frequency Distribution and Size Inequality of the First-Order Roots of Fraxinus mandschurica in a Mixed Mature Pinus koraiensis Forest
Source: Front Plant Sci. 2017 Sep 26;8:1691. doi: 10.3389/fpls.2017.01691 (PMC5622955; doi:10.3389/fpls.2017.01691)
Supplement: Supplementary file 1 [file DataSheet1.docx]

**Supplementary information**

| Factors | DF | FL | FD | FA | SL | SD | SA | BI |
| --- | --- | --- | --- | --- | --- | --- | --- | --- |
| N | 1 | 4.096^*^ | 0.970^ns^ | 3.982^*^ | 0.000^ns^ | 1.503^ns^ | 0.096^ns^ | 1.143^ns^ |
| W | 1 | 10.059^**^ | 0.153^ns^ | 9.371^**^ | 0.026^ns^ | 8.522^**^ | 1.332^ns^ | 0.002^ns^ |
| NW | 1 | 22.416^***^ | 22.166^***^ | 33.780^***^ | 6.099^*^ | 0.006^ns^ | 1.745^ns^ | 6.576^*^ |

TABLE S1 | Effects of nitrogen addition (N), rainfall reduction (W) and their interaction (NW) on first-order root length (FL), diameter (FD) and area (FA), on second-order root length (SL), diameter (SD) and area (SA), and on branching intensity (BI), analyzed using two-way ANOVAs. DF: degrees of freedom. Significance level: ns: not significant (*p* > 0.05); ^*^*p* < 0.05; ^**^*p* < 0.01; ^***^*p* < 0.001. *F*-values are given.


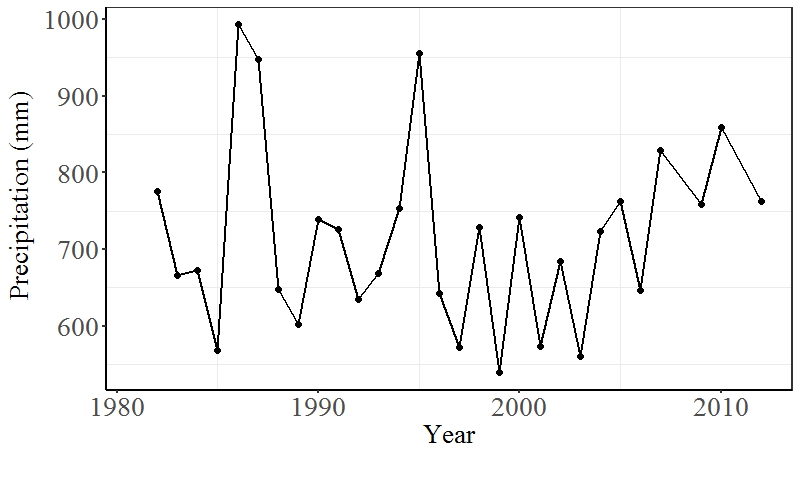


FIGURE S1 | The annual mean precipitation (1982-2012) in the mixed mature *Pinus koraiensis* forest on Changbai Mountain, China.


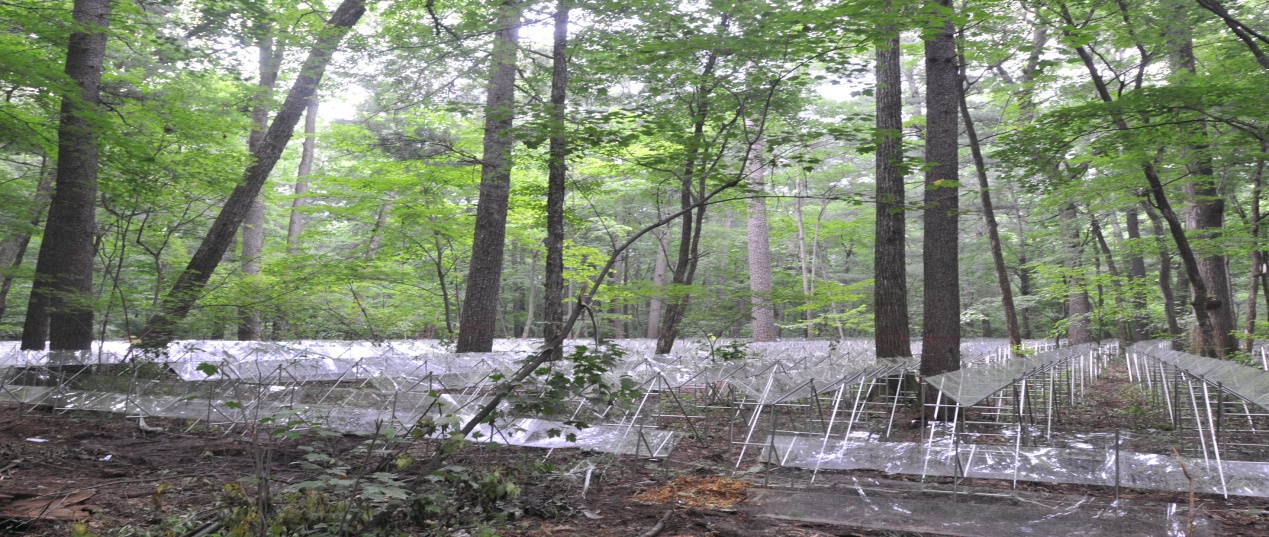


FIGURE S2 | The rainfall reduction facility in the mixed mature *P. koraiensis* forest on Changbai Mountain, China (photo credit: Cunguo Wang).
